# Miscellany

**Published:** 1905-09

**Authors:** 


### MISCELLANY.

BATTLE & COMPANY, Saint Louis, have just issued the sixth of their series of twelve illustrations, of the Intestinal parasites, and they will send them free, to physicians, on application.

CIVIL SERVICE EXAMINATIONS FOR THE STATE AND COUNTY SERVICE.—The State Civil Service Commission has announced a general examination to be held September 9. Among the positions included in this examination are those of pupil nurse, Erie County Hospital, \$120 to \$180 and maintenance; woman officer, state institutions, \$25 a month and maintenance. The last day for filing applications for these positions is September 4; application forms and detailed information may be obtained by addressing the chief examiner of the commission at Albany.

THE *Medico-Pharmaceutical Journal,* of New York, has removed its office of publication to 45 West 128th street.

